# The *NQO1* allelic frequency in hindu population of central India varies from that of other Asian populations

**DOI:** 10.4103/0971-6866.73403

**Published:** 2010

**Authors:** Sher S. Parihar, U. K. Chauhan

**Affiliations:** School of Environmental Biology, Awdhesh Pratap Singh University, Rewa 486 003, Madhya Pradesh, India

**Keywords:** Cancer, *NQO1*, polymorphism

## Abstract

**CONTEXT:**

The enzymes encoded by the polymorphic genes NAD (P) H: quinone oxidoreductase 1 (*NQO1*) play an important role in the activation and inactivation of xenobiotics. This enzyme has been associated with xenobiotic related diseases, such as cancer, therapeutic failure and abnormal effects of drugs.

**AIM:**

The aim of the present study was to determine the allelic and genotypic frequencies of NQO Hinf I polymorphisms in a Hindu population of Central India.

**SETTINGS AND DESIGN:**

Polymorphisms of *NQO1* were determined in 311 unrelated Hindu individuals.

**MATERIALS AND METHODS:**

Polymerase chain reaction- Restriction Fragment Length Polymorphism (PCR-RFLP) analysis in peripheral blood DNA for *NQO1* Hinf I polymorphism was used in 311 unrelated Hindu individuals.

**STATISTICAL ANALYSIS:**

Allele frequencies were calculated by direct counting. Hardy Weinberg Equilibrium was evaluated using a Chi-square goodness of fit test.

**RESULTS:**

The observed allelic frequency was 81% for C (wild) and 19% for T (mutant) in the total sample.

**CONCLUSIONS:**

The allelic frequency of “C” was higher than in other Asians (57%), but similar to Caucasians (81%). The genotype distributions for Hinf I polymorphisms were in Hardy-Weinberg equilibrium.

## Introduction

Phase I enzyme DT-diphorase [NAD (P) H: quinone oxidoreductase 1 (*NQO1*)] converts toxic benzoquinone into hydroquinone in an obligate two-electron reduction reaction and is a key enzyme in polycyclic hydrocarbon metabolism in humans.[1] This reaction competes with one-electron reduction reactions by cytochrome *P*-450, producing the semiquinones, which generate free radicals and reactive oxygen species via redox cycling. The *NQO1* is capable of maintaining these quinones in their reduced form, and therefore, detoxifies them.[2,3]

Two polymorphic variants of *NQO1* have been identified: a C-to-T change at nucleotide 609 yields a serine substitution and a T-to-C change at nucleotide 464 results in tryptophan replacement of arginine. The C 609 T effectively inactivates the enzyme due to decreased catalytic activity and stability of *NQO1* protein.[4,5] So, it is supposed that *NQO1* has a crucial role in cancer susceptibility. Among Northern Europeans and Caucasian Americans, the gene frequency is about 79% for the wild-type allele and 21% for the mutated allele.[6,7] The frequency of the mutated allele is known to be slightly higher among African Americans and considerably higher among Hispanics and Asians.[8] *NQO1* enzyme activity is found to be normal in individuals with wild-type alleles. It is variably reduced in individuals who are heterozygotes for the polymorphism.[9] The *NQO1* protein and its activity are absent in those who are homozygous for the point mutation.[10] It is reported that inhibition of *NQO1* activity by dicoumarol induces degradation of p53. This indicates that *NQO1* plays a role in p53 stabilization, which has a crucial role in cell cycle regulation.[11] In various studies, *NQO1* null alleles have shown to be associated with benzene related leukemia,[12] cancer of lung,[13] and colon.[14] A study suggested that *NQO1* can modulate the susceptibility for breast cancer[15] Chang Gun Cho and coworkers concluded that *NQO1 139Arg* alleles were associated with tobacco dose-dependent increase in risk of head and neck squamous cell carcinoma (HNSCC), and *NQO1* genotype polymorphisms may play an important role in the development of smoking-related HNSCC.[16] However, Hongwei Chen and coworkers have found no relation between *NQO1* null allele and lung cancer.[17] But this does not render the significance of *NQO1*. It is also a highly inducible enzyme. Synthetic antioxidants such as butyrate hydroxyanisole and extracts of cruciferous vegetables, including broccoli, have been shown to be potent inducers of *NQO1*. This inducibility has led to the suggestion that *NQO1* can play an important role in cancer chemoprevention and therapy.

## Materials and Methods

Blood was drawn from the 311 normal, healthy, unrelated Hindu individuals [Table 1]. All the subjects were the regular residents of the Vindhyan region, the heart land of India. This name originated from Vindhya Range, which is a range of older rounded mountains and hills in the west-central Indian subcontinent, which separates the Indian subcontinent into northern India (the Indo-Gangetic plain) and southern India. The study was approved by the ethical council of APS University, Rewa. Blood samples were drawn by intravenous injection and 3–5 ml of blood was collected in ethylenediamine tetraaceticacid (EDTA) containing bowls and was stored at −20°C until use. Genomic DNA was extracted from whole blood by a slight modification of salting out procedure described by Miller and coworkers.[18] A C-to-T change at nucleotide 609 in *NQO1* genes yields a serine substitution, which creates a Hinf I cleavage site. Primer was designed to amplify the 172 bp sequence to study this polymorphism. The oligonucleotide sequences (primers) used were those described by Jianhui Zhang *et al*.[19] For each DNA sample, 25 μl of polymerase chain reaction (PCR) mixture was prepared containing 5 μl template DNA (final concentration 100–200 ng/μl), 2.5 μl of 10× Taq polymerase buffer (10 mM Tris HCl, pH 8.8, 50 mM KCl, 1.5 mM MgCl2, 0.01% gelatin, 0.005% Tween-20, 0.005% NP-40; final concentration 1×; Genetix Biotech Asia Pvt. Ltd., New Delhi, India), 0.5 μl of 10 mM dNTPs (Banglore Genei, Bangalore, India), 0.5 μl of 25 pmol/μl of forward and reverse primers specific for *NQO1* genes, 0.5 μl of 5 U/μl of Taq DNA polymerase (final concentration 1 U; Genetix Biotech Asia Pvt. Ltd., India) and sterile water to make up the volume of reaction mixture to 25 μl. Thermal profile used for the amplification of desired segment of gene was as follows: initial denaturation at 95°C for 5 min and 30 cycles of denaturation at 94°C for 45 sec, annealing at 62.9°C for 45 sec and extension at 72°C for 1 min, followed by final extension at 72°C for 07 min. PCR products were separated on 2% agarose gel (2% w/v, Sigma, Signa Adrich, Bangalore, India) using a 100-bp molecular weight (MW) marker to confirm the PCR product size of 172 bp.

**Table 1:** Clinical features of study population

Clinical features	Males	Females
Total no.	136	175
Sex ratio (%)	43.73	56.27
Age	33.43 ±13.96	
Mean ± SD	32.00	
Age range	11–73	

For restriction digestion of the C-to-T substitution at nucleotide 609 non-coding region, which creates an Hinf I restriction enzyme cleavage site, the reaction mixture included 0.2 μl of 10,000 U/ml Hinf I restriction enzyme (final concentration 2.5 U), 2.5 μl of 10× GENAI buffer c (final concentration 1×; 50 mM potassium acetate, 20 mM Tris acetate, 10 mM magnesium acetate, 1 mM Di This Thretal (DTT), pH 7.9), 10.0 l of PCR product and 10 μl of sterile water. Reaction was incubated for 24 hours at 37°C for complete digestion. Ten microliters of digested PCR product was loaded on 2% agarose gel. Electrophoresis was done at 80 V in 1× Tris-borate EDTA buffer (89 mM Tris pH 7.6, 89 mM boric acid, 2 mM EDTA pH 8.0). A 100-bp gene DNA ladder (Genetix, India) was run concurrently as a molecular weight marker. The gel was than stained with ethidium bromide (10 mg/ml). The products were visualized using an ultraviolet transilluminator. The gel picture was captured using a digital camera and gel documentation software (Vilber Lourmate, Cedex I, France).The *NQO1* wild-type allele shows a 172-bp PCR product resistant to enzyme digestion, whereas the null allele shows a 131-bp and 41-bp band in 2% agarose gel.

## Results

**Figure 1:**
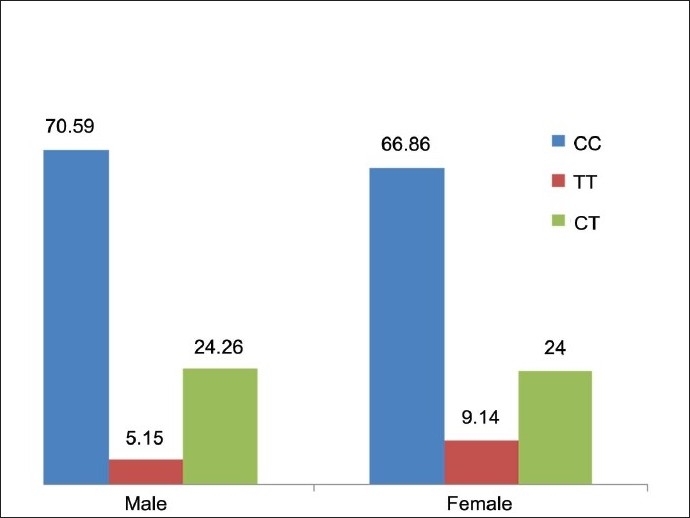
*NQO1* Genotype distribution among Hindu Male and Female Population (In %)

**Figure 2:**
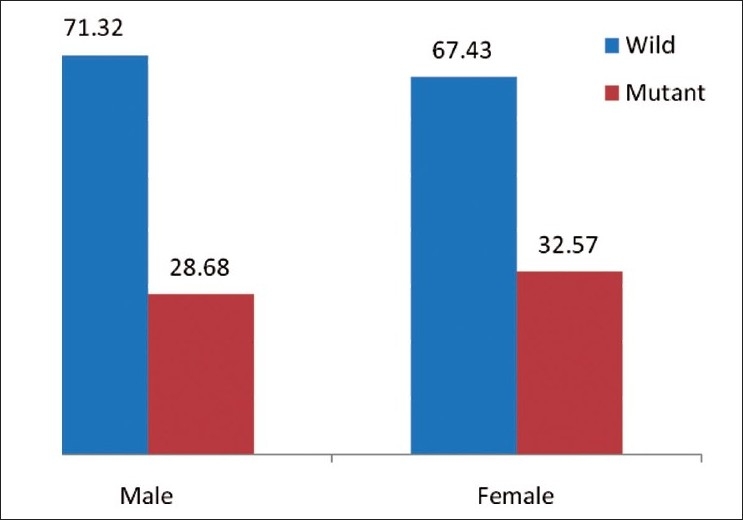
*NQO1* Allelic distribution in Hindu Male and Female Population (In %)

In our investigation, the polymorphism of *NQO1* genes was studied by PCR-RFLP. The C-to-T substitution at nucleotide 609 creates the restriction site for Hinf I. The PCR products of wild-type allele (CC) were 172 bp long and were undigestable by Hinf I at standard condition. Heterozygous condition (CT) appeared as 172, 131 and 41 bp long DNA fragments were produced after digestion with Hinf I. The null alleles (TT) were detected as the 131 and 41 bp long fragments were produced after digestion with Hinf I at standard condition. The genotype frequency of CC, TT and CT genotypes scored 70.59, 5.15 and 24.24%, respectively, in males. In females, is the percentages were CC = 66.86%, TT = 9.14% and CT = 24% [Table 2 Figure 1] The association of genotype frequency distribution in both the groups (Hindu males and Hindu females) was studied and it was observed that there were no statically significant differences as the χ^2^ and *P* values were 0.3768 and 0.5393, respectively. The distribution of C and T alleles in both the groups was C = 71.32% and T = 28.68% in male Hindus and C = 67.43% and T = 32.57% in females [Table 2 Figure 2]. The association between allelic distribution in both the groups was studied by Chi^2^(with Yates correction) test and values obtained were χ^2^ = 1.810 and *P* = 0.4046. All the values were nonsignificant, which showed that the frequency distribution of C and T alleles in both the groups was statically not Significant [Table 2].

**Table 2:** Distribution of *NQO1* genotypes and allele frequencies and their association between study groups

Category	No.	Wild allele C	Mutant allele T	CC	TT	CT
Male	136	No. (%) 97 (71.32)	No. (%) 39 (28.68)	No. (%) 96 (70.59)	No. (%) 07 (5.15)	No. (%) 33 (24.26)
Female	175	118 (67.43)	57 (32.57)	117 (66.86)	16 (9.14)	42 (24)
Chi square		1.810 NS			0.3768 NS	
*P* value (with Yates correction)		0.4046 NS			0.5393 NS	

## Discussion

*NQO1* is a key enzyme in carcinogen metabolism and its allelic frequency varies by ethnicity. The variable frequency of *NQO1* makes some populations more susceptible for getting cancerous disease due to the inability to detoxify the carcinogen by null allele. This fact led us to study the frequency of *NQO1* in a population of Vindhyan region (Central India). Our study pointed at some important features of Hindu population, which were unknown until this study. Our results showed that the frequency of *NQO1* in Hindu population was different from other Asian populations and slightly similar to Caucasian population.

The *NQO1 Hinf* I genotype frequency of our population was not statically different with that reported in Maharashtrian Indians where CC = 52.3%, CT = 42.48% and TT = 5.18%.[20] But an increase in heterozygosity was observed in Maharashtrian Indians than in our population. It could be due to random mixing of various ethnic groups in Maharashtrian population in the modern age. But increase in heterozygosity surprised us because the marriage between the close communities is common among the Maharashtrians.

**Figure 3:**
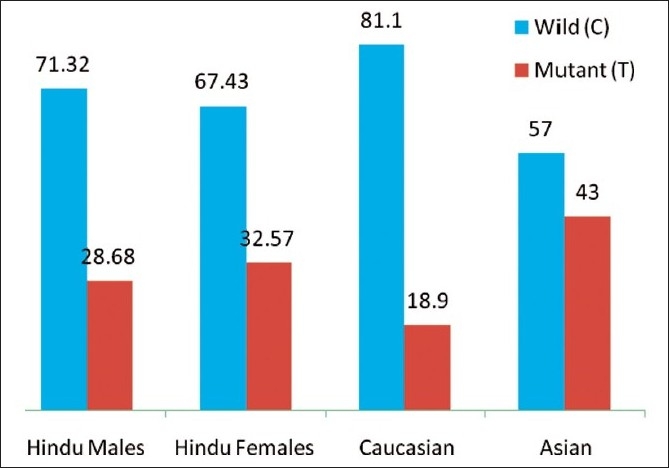
*NQO1* allelic distribution in Caucasian, Asian and Hindu Male, Female populations (In %)

Our results showed that the frequencies of different polymorphs of *NQO1* genes are statically distinct in our population than in Caucasian and other Asian populations, although the differences are much evident between our population and other Asian populations [Table 3 Figure 3]. The frequencies observed in our population also did not match with the Caucasian, Chinese and Koreans, where the frequencies reported were CC = 79%, CT = 16% and TT = 05% in Caucasian,[22] CC = 34%, CT = 49.7% and TT = 16.3% in Chinese,[23] and CC = 94.5%, CT = 5.2% and TT = 0.3% in Koreans.[24] This showed that these populations are distinct from our study population. The Swedish population showed a slight difference with our population, where the frequencies reported were CC = 69.4%, CT = 28.9% and TT = 1.7%.[25] But the distribution of TT (mutant) allele was less in Swedish than in our population. The genotype frequencies of our population also matched with the population of different Iranian ethnic groups[26] on comparison, where the recorded frequencies were CC = 59.5%, CT = 31% and TT = 9.5% in Fars, CC = 64%, CT = 24% and TT = 12% in Mazzandarani and CC = 69%, CT = 28.6%, TT = 2.4% in Turks. But an increase in TT (mutant) genotype was observed in our population and in two Iranian ethnic groups (Fars and Mazzandarani) than in Turks and other world populations. The results are in agreement with the phenomena of common ancestry of Indian and Iranian population.

**Table 3:** *NQO1* allelic frequency in Caucasian, Asian and Hindu male, female population (%)

Population	C	T	References
Hindu males	71.32	28.68	Present study
Hindu females	67.43	32.57	Present study
Caucasian	81.1	18.9	Kiyohara *et al*.[21]
Asian	57	43	

The allelic distributions are found to be in accordance with other findings in Caucasian population (C = 81.1% and T = 18.9%),[27] as there were no significant differences observed. Significant differences were seen when our findings were compared with that of others in various Asian populations, where the frequency of C allele was reported to be 57% and that of the T allele was 43%, as calculated in a previous finding by Kiyohara *et al*.[21] This difference was also evident when our results were compared with the individual findings of Choi *et al*.[28] in Korean and Fowke *et al*.[29] in Chinese populations, where the frequencies were recorded as C = 58.5% and T = 41.5% in Korean and frequency of C allele was 59.3% and T allele was 40.7% in Chinese. These results did not surprise us because in calculation of *NQO1 Hinf* I allele, Kiyohara *et al*. did not include the frequencies of Indian population and their findings were solely based on the results from Korean, Chinese and Japanese populations, which are totally distinct from our population and belong to different races. This finding suggests that the Indian population cannot be correlated with the rest of the Asian population.

## References

[1] Lind C, Cadenas E, Hochstein P, Ernster L (1990). DT-diaphorase: Purification, properties, and function. Methods Enzymol.

[2] Kim K, Suk H (1999). Reduction of nitrosoarene by purified NAD (P) H-quinone oxidoreductase. J Biochem Mol Biol.

[3] Dinkova-Kostova AT, Talalay P (2000). Persuasive evidence that quinone reductase type 1 (DT-diaphorase) protects cells against the toxicity of electrophiles and reactive forms of oxygen. Free Radic Biol Med.

[4] Jaiswal AK (1991). Human NAD (P) H: Quinone oxidoreductase (*NQO1*) gene structure and induction by dioxin. Biochemistry.

[5] Jaiswal AK (2000). Regulation of genes encoding NAD (P) H: Quinone oxidoreductase. Free Radic Biol Med.

[6] Wiencke JK, Spitz MR, McMillan A, Kelsey KT (1997). Lung cancer in Mexican-Americans and African-Americans is associated with the wild-type genotype of the NAD (P) H: Quinone oxidoreductase polymorphism. Cancer Epidemiol Biomarkers Prev.

[7] Traver RD, Siegel D, Beall HD, Phillips RM, Gibson NW, Franklin WA (1997). Characterization of a polymorphism in NADP (H): Quinone oxidoreductase (DT diaphorase). Br J Cancer.

[8] Kelsey KT, Ross D, Traver RD, Christiani DC, Zuo ZF, Spitz MR (1997). Ethnic variation in the prevalence of a common NAD (P) H: Quinone oxidoreductase polymorphism and its implications for anti-cancer chemotherapy. Br J Cancer.

[9] Ross D, Traver RD, Siegel D, Kuehl BL, Misra V, Rauth AM (1996). A polymorphism in NAD (P) H: Quinone oxidoreductase (*NQO1*): Relationship of a homozygous mutation at position 609 of the *NQO1* cDNA to *NQO1* activity. Br J Cancer.

[10] Traver RD, Rothman N, Smith MT, Yin SY, Hayes RB, Li GL (1996). Incidence of a polymorphism in NAD (P) H: Quinone oxidoreductase (*NQO1*). Proc Am Assoc Cancer Res.

[11] Asher G, Lotem J, Kama R, Sachs L, Shaul Y (2002). *NQO1* stabilizes p53 through a distinct pathway. Proc Natl Acad Sci USA.

[12] Smith MT (1999). Benzene, *NQO1*, and genetic susceptibility to cancer. Proc Natl Acad Sci USA.

[13] Kiyohara C, Yoshimasu K, Takayama K, Nakanishi Y (2005). *NQO1*, MPO, and the risk of lung cancer: A Huge review. Genet Med.

[14] Begleiter A, Hewitt D, Maksymiuk AW, Ross DA, Bird RP (2006). A NAD (P) H: Quinone Oxidoreductase 1 Polymorphism Is a Risk Factor for Human Colon Cancer. Cancer Epidemiol Biomarkers Prev.

[15] Fowke JH, Shu XO, Dai Q, Jin F, Cai Q, Gao YT (2004). Oral Contraceptive Use and Breast Cancer Risk: Modification by NAD (P) H: Quinone Oxoreductase (*NQO1*) Genetic Polymorphisms. Cancer Epidemiol Biomarkers Prev.

[16] Cho CG, Lee SK, Nam SY, Lee MS, Lee SW, Choi EK (2006). Association of the GSTP1 and *NQO1* Polymorphisms and Head and Neck Squamous Cell Carcinoma Risk. J Korean Med Sci.

[17] Chen H, Lum A, Seifried A, Wilkens LR, Le Marchand L (1999). Association of the NAD (P) H: Quinone Oxidoreductase 609C3T Polymorphism with a Decreased Lung Cancer Risk. Cancer Res.

[18] Miller SA, Dykes DD, Polesky HF (1988). A simple salting out procedure for extracting DNA from human nucleated cells. Nucleic Acids Res.

[19] Zhang J, Schulz WA, Li Y, Wang R, Zotz R, Wen D (2003). Association of NAD (P) H: Quinone oxidoreductase 1 (*NQO1*) C609T polymorphism with esophageal squamous cell carcinoma in a German Caucasian and a northern Chinese population. Carcinogenesis.

[20] Saravana DS, Vinayagamoorthy N, Agrawal M, Biswas A, Biswas R, Naoghare P (2008). Distribution of detoxifying genes polymorphism in Maharastrian Population of CentralIndia. Chemosphere.

[21] Chikako K, Kouichi Y, Koichi T, Yoichi N (2005). *NQO1*, MPO, and the risk of lung cancer: A HuGE review. Genet Med.

[22] Toncheva DI, Von Ahsen N, Atanasova SY, Dimitrov TG, Armstrong VW, Oellerich M (2004). Identification of *NQO1* and GSTs genotype frequencies in Bulgarian patients with Balkan endemic nephropathy. J Nephrol.

[23] Zhang J, Schulz WA, Li Y, Wang R, Zotz R, Wen D (2003). Association of NAD (P) H: Quinone oxidoreductase 1 (*NQO1*) C609T polymorphism with esophageal squamous cell carcinoma in a German Caucasian and a northern Chinese population. Carcinogenesis.

[24] Cho CG, Lee SK, Nam SY, Lee MS, Lee SW, Choi EK (2006). Association of the GSTP1 and *NQO1* Polymorphisms and Head and Neck Squamous Cell Carcinoma Risk. J Korean Med Sci.

[25] Alexandrie AK, Nyberg F, Warholm M, Rannug A (2004). Influence of CYP1A1, GSTM1, GSTT1, and *NQO1* Genotypes and Cumulative Smoking Dose on Lung Cancer Risk in a Swedish Population. Cancer Epidemiol Biomarkers Prev.

[26] Biramijamal F, Sanati MH, Banoei MM, Bayat B, Arjmand S, Farhud D (2006). Genetic polymorphism analysis of NAD (P) : Quinone oxidoreductase 1 in different Iranian ethnic groups. Curr Sci.

[27] Chikako K, Kouichi Y, Koichi T, Yoichi N (2005). *NQO1*, MPO, and the risk of lung cancer: A HuGE review. Genet Med.

[28] Choi JY, Lee KM, Cho SH, Kim SW, Choi HY, Lee SY (2003). CYP2E1 and *NQO1* genotypes, smoking and bladder cancer. Pharmacogenetics.

[29] Fowke JH, Shu XO, Dai Q, Shintani A, Conaway CC, Chung FL (2003). Urinary isothiocyanate excretion, brassica consumption, and gene polymorphisms among women living in Shanghai, China. Cancer Epidemiol Biomarkers Prev.

